# Central place foraging in an ectotherm and the long-term liability of selecting the “wrong” central place

**DOI:** 10.1093/beheco/araf062

**Published:** 2025-05-29

**Authors:** Terry J Ord, Katrina Blazek

**Affiliations:** Evolution and Ecology Research Centre, and the School of Biological, Earth and Environmental Sciences, University of New South Wales, Kensington NSW 2052, Australia; School of Population Health, University of New South Wales, Kensington NSW 2052, Australia

**Keywords:** climate change, ectotherm, environmental stress, nest site, temperature-dependent activity

## Abstract

Shelter provided by a fixed refuge like a burrow or nest has obvious advantages for central place foragers: the energy and time required to construct the refuge is expected to be offset by improvements in survival and reproduction. This assumes the refuge is positioned appropriately in the environment to access food and other resources, and that the environment itself remains stable over time. We investigated the long-term liability of refuge placement and adverse environmental change on an iconic central place forager, the Australian meat ant (*Iridomyrmex purpureus*). We measured the consequences of nest location on the thermal conditions experienced at the nest and how those conditions influenced the opportunity to forage and defend the nest from predation. These data were combined with direct field observations of foraging effort and changes in nest size obtained from over a decade of study. Getting the location of a nest wrong had a lasting impact on the growth of a nest and colonies were unable to compensate for subsequent time restrictions on activity through changes in behavior. Our data suggests that central place foragers relying on the long-term occupancy of a fixed refuge are especially vulnerable to environmental change. Unless these species compensate through changes in behavior or construct a new central refuge in an area outside of the zone of impact, the increasing frequency and severity of environmental change occurring with the climate crisis could increase the risk of local extinction.

## Teaser

Long-lived ant colonies that get the position of nests wrong are less likely to survive prolonged environmental change. A decade of monitoring shows Australian meat ants must place their nests carefully but may still be vulnerable to future changes in environmental conditions because of the long-term occupancy of nests. This suggests other long-lived animals who are also tied to the location of a nest or burrow are susceptible to local extinction, especially from climate change.

## Introduction

Central place foragers are species that repeatedly return to some preferred site following periodic bouts of foraging. These ‘central places’ might be a reef from which a fish ventures out to feed amongst the surrounding seagrass (DiFiore et al. 2019; Madin et al. 2022) or a favored basking site from which a lizard surveys the surrounding environment for insect prey (Stamps 1977; Chase 1998). In many cases, the animals themselves invest heavily in the construction of the central place in the form of a refuge, such as a nest or burrow. There are adaptive benefits for reproduction and survival from this investment through the protection a refuge provides against predation (Manicom et al. 2008; Orrock et al. 2013; Deeming 2023), parasitism (Scott-Baumann and Morgan 2015; Deeming 2023) and fluctuations in environmental conditions (eg daily or seasonal; Ultsch 2006; Akresh et al. 2017; Lowney et al. 2020; Deeming 2023). Nevertheless, repeatedly returning to a central place can compromise an animal’s general mobility (eg Pichegru et al. 2010). As the time and energy required to construct a central refuge increases, individuals are presumably less likely to establish a new base at an alternative site. The placement of the refuge is therefore paramount for foragers in determining both the immediate and future accessibility of food and other resources (Holway and Case 2000; Bakker et al. 2005; Wakefield et al. 2014; Calderón-Capote et al. 2020; Aarts et al. 2021; McHuron et al. 2023) as well as the refuge’s exposure to abiotic conditions (Dziadzio et al. 2016; Bodensteiner et al. 2023; Ord 2023).

The compromise to mobility from occupying a central place might ultimately prove detrimental to a forager if conditions change in the environment (Pichegru et al. 2010). For example, central place foragers are vulnerable to the progressive depletion of resources around their refuges (Chase 1998; Elliott et al. 2009; Patenaude-Monette et al. 2014; Calderón-Capote et al. 2020). This can be offset to an extent with greater forays out into the environment as depletion “halos” increase in size (Pichegru et al. 2010; Weber et al. 2021; Madin et al. 2022; Patterson et al. 2022; see also Gordon 2002; Gordon et al. 2008), at the expense of energy, time and exposure to predation and other hazards (Patenaude-Monette et al. 2014; Vance et al. 2021). While refuges provide protection against short-term abiotic stressors such as fluctuations in weather, refuges are unlikely to mitigate the community-wide impacts that result from prolonged or severe environmental change (eg Pichegru et al. 2010). The adoption of a central place foraging strategy therefore has a range of potential ecological challenges associated with it, from the appropriate placement of a refuge to the long-term residency of that central place when conditions in the environment change.

Much of our understanding of central place foragers comes from theoretical models focused on defining its putative benefits (eg Aarts et al. 2021; Robinson et al. 2022; McHuron et al. 2023), or empirical studies reporting spatial patterns of resources and its impacts on forager behavior (eg Orgeret et al. 2021; Gicquel et al. 2022; Iorio-Merlo et al. 2022). We know little about the short-term or long-term liabilities associated with central place foraging, and how species might cope with the immediate and future challenges associated with the placement of a central refuge and its subsequent long-term occupancy. This gap likely reflects the difficulty in quantifying the benefits and costs of occupying a particular central place relative to other sites in the environment, and then monitoring the consequences of remaining at that site over long periods of time (eg months to years). It would appear, though, that central place foragers limited in their capacity to migrate to new areas are at risk of local extinction from environmental change, which is concerning given the increased frequency and severity of droughts, floods and bushfire currently occurring from the climate crisis (Karl and Trenberth 2003; Rahmstorf and Coumou 2011; Coumou and Rahmstorf 2012; Screen and Simmonds 2014;  Jain et al. 2021). In this study, we examined the legacy of occupying a central place on the activity and survival of an iconic central place forager in an environment that has suffered severe drought.

Ants are classic examples of central place foragers, which invest in the construction of a nest from which they forage out into the landscape. In many ant species, the central refuge of the nest can be moved by the colony to track shifting resources in the environment (eg Argentine ants: Hui and Pinter-Wollman 2014; harvester ants; Pinter-Wollman and Brown 2015) or escape seasonal changes effecting the microclimate of nests (Elias et al. 2005; reviewed by McGlynn 2012). In some species, however, the size or complexity of the nest (eg see O’Fallon et al. 2023) effectively anchors colonies to a single location for extended periods of time because of the substantial investment involved in building the nest (Anderson 1995). In particular, colonies of the iconic Australian meat ant (*Iridomyrmex purpureus*) can be extremely large (eg millions of individuals) and build enormous ground nests that remain active for years. It is not unusual for some nests to be decades old (Greenslade 1973; Ord 2023). This longevity logically places a premium on the appropriate positioning of these central refuges, while similarly constraining the capacity of colonies to shift to alternative locations if conditions should change over time (ie the budding of new nests is rare—see Greaves and Huges 1974; Greenslade 1975; Ord 2023).

**Figure 1.**
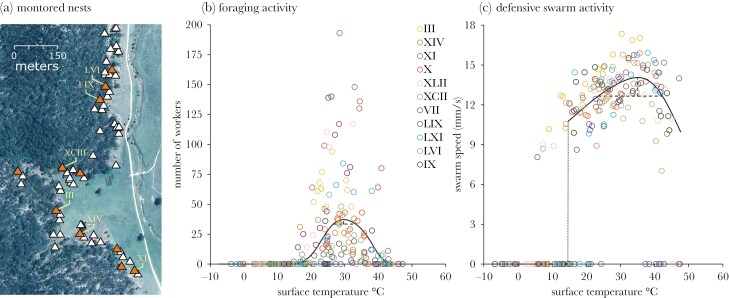
(a) Eleven meat ant nests were studied to compute temperature-dependent performance curves of (b) foraging and (c) defensive swarm activity. Of the eleven nests (highlighted in orange in a), six had permanent temperature probes secured to the surface of the nest (labeled by roman numerals) that recorded surface temperature at 30 min intervals for at least two years. Temperatures at which maximum and optimal activity (90% of from this maximum) were computed to occur are defined by the dashed lines (b, c). Data points are color coded by nest ID.

For over a decade, we have monitored a large population of meat ants in the NSW Central Tablelands in Australia. Observation of nest positions suggests meat ant colonies select sites that balance shade and sun exposure to maintain the nest within a certain thermal window or niche. The apparent result is the clustering of nests along the boundary of a sclerophyll woodland (providing shade) and open pasture (providing sun exposure; Fig. 1a). Maintaining a nest within a particular temperature window is important for the incubation and development of eggs and larvae within the nest (Kipyatkov and Lopatina 2002; Abril et al. 2010), but will also impact the capacity of workers to be active outside the nest (eg Bernstein 1979; Jayatilaka et al. 2011; Stuble et al. 2013; Roeder et al. 2022; see also Parr and Bishop 2022). This activity includes workers scavenging out from the nest for dead insects, other carrion, seeds and other plant material, and the maintenance of cleared trails to eucalyptus trees, where workers harvest sugars secreted from aphids residing in the trees (Bottinelli et al. 2015; see also Sagata and Gibb 2016). Anti-predator defence of the nest consists of large swarms of biting workers surging out of the nest and blanketing its surface and attacking the source of any perceived threat. This defensive swarm is the primary defence against predation by echidnas (eg van Wilgenburg and Elgar 2007). If nest position is a strong determinant of the range of temperatures experienced by workers, then it follows that nest position should dictate the ability of a colony to adequately forage and defend the nest against predation. Moreover, the extent a colony can subsequently compensate for a poorly chosen nest site, or otherwise changes in the temperature conditions experienced at the nest, will be critical for a colony’s capacity to mitigate or buffer against adverse environmental change over the long-term.

In 2017 through to early 2020, the monitored population of meat ants in the NSW Central Tablelands experienced the worst drought in Australian recorded history (bom.gov.au). The severity of this event provided an ideal opportunity to tease out the impact of nest location on colony survival resulting from environmental change, and especially within the broader context of how the increasing frequency and severity of such adverse events from the climate crisis will likely impact this iconic species. The toll of the drought on the surrounding environment was extraordinary, with extensive dieback of ground vegetation and thinning of tree canopy cover. The establishment of new nests by meat ants is usually rare (Greaves and Hughes 1974; Greenslade 1975) and this population had been stable for many years prior to 2017 (Ord 2023). With the onset of drought, however, many meat ant colonies attempted to establish satellite nests through an unprecedented surge in nest budding (Ord 2023). This seemed to be an attempt by colonies to move their nests into areas with more favorable microclimate conditions (eg into the tree line to maintain an appropriate thermal niche). The success of this strategy was variable, with an unusually high number of subsequent nest abandonments. Those nests that had more feeding trails, on average, were also less susceptible to extinction (Ord 2023). It was unclear, however, whether this link between colony survival and foraging activity was the outcome of a colony’s success in tracking the shifting microclimate conditions during drought (through nest budding), or its ability to compensate for changes in resource availability by increasing foraging effort more generally (independent of whether colonies attempted to relocate through budding).

In this study we quantified: (i) the abiotic conditions experienced at central refuges (meat ant nests) positioned in different locations within the same environment; (ii) how those abiotic conditions impacted the opportunity for foraging and defence; (iii) the capacity of the meat ant colony to behaviorally compensate for constraints on that activity; and (iv) how the occupancy of a central refuge impacts nest growth following acute and long-lasting environmental change. Our broad goal was to provide rare data on the extent to which the long-term occupancy of a central refuge has the capacity to exacerbate or buffer the risk of local extinction in central place foragers.

## Materials and methods

Our study was conducted in several parts. First, we estimated the temperature-dependent performance curves of foraging activity and defensive swarms across 11 meat ant nests from separate colonies at hourly intervals from dawn through to dusk on a single day in mid-summer and mid-winter. Second, for six of these 11 nests, we recorded nest surface temperatures at 30-min intervals for at least two years to assess how nest location determined the microclimate conditions experienced by the colony across seasons and years. Third, these nest surface temperature data were combined with the temperature-dependent performance curves to compute the number of daily hours available for foraging and defensive swarms for each of the six nests. Fourth, the extent to which nests compensated for temperature-dependent restrictions on activity was determined by assessing whether colonies increased foraging effort (indexed by the number of foraging and tree trails maintained) as the number of hours available for activity decreased. Finally, the long-term impact of nest location—and its associated influence on worker activity—was evaluated by monitoring annual changes in nest size over 11 years (2015 to 2025).

Full methodological details are given in the Supporting Information, with the sections below providing a general summary.

### Study system

The population of meat ants has been intensively monitored annually since 2015, and informally since 2010, and is located near the locality of Wollar in New South Wales, Australia. The size of the population in a typical year ranges from 1 to 2 nests per 100 m^2^ (or 30 to 50 nests in total; Fig. 1a) with the establishment of new nests in being less than 0.2 nests per 100 m^2^ (< 5 nests per year; Ord 2023). Surveys of every nest were exhaustively carried out each year during the Australian summer (February to March). Each nest has been mapped with GPS and permanently labeled with a metal tag. Nest size is recorded using counts of entrance holes (see Supporting Information), and activity indexed by the total number of active foraging trails, tree trails, and connection trails to other nests. Active trails were defined as any trail in which a constant traffic of workers was seen moving along the trail.

### Estimating temperature-dependent performance curves

Eleven nests were selected to reflect a broad range in size (median: 32 entrance holes; range 6 to 58) and locations expected to vary in level of shading (Fig. 1a). Historic data from annual surveys of the population were used to confirm each nest belonged to a separate meat ant colony (ie have never been connected by a common trail). Foraging activity for each nest was measured by counting the number of workers moving along a known tree trail (see Supporting Information for why tree trails were favoured over the more temporary foraging trails). While a mega-nest can have as many as 13 tree trails active at once, most typically have only one (see Ord 2023). This was the case for the 11 nests examined in the current study. Nevertheless, the opportunity was taken to return to the nest each hour to confirm no new trail had been established. For the single tree trail observed for each nest, workers moving along this trail were counted at hourly intervals from dawn through to dusk, on a single day in mid-summer (January) and mid-winter (July) to capture the broad range of temperatures experienced by workers (ie summer captures the upper temperatures of the performance curve, while winter captures the lower temperatures of the performance curve; four nests were sampled in 2017 (nests III, XIV, LXIV, and X) with the remaining seven in 2018). Defensive swarm activity was measured as swarm speed by computational motion analysis (Peters et al. 2002) using video recorded at hourly intervals from dawn to dusk at the same time and on the same summer and winter days as foraging activity (previous paragraph). Swarms were induced by scraping the surface of the nest three times in a systematic manner using a stick. This simulated a predator attack (eg disturbance by an echidna). Meat ant swarms operate as an effective defence by flooding the surface of the nest with workers who then converge on and repeatedly bite the source of disturbance. We used the speed of the induced swarm to estimate defence performance because it measured both how rapidly workers exited the nest and how fast they would converge and attack a perceived threat. Substrate surface temperature was measured at the center of the trail or nest and at the same time that foraging and swarm activity were measured. In addition to quantifying the activity of meat ants as a function of temperature, we also evaluated whether activity was similarly dependent on ambient light, time of day or wind. Meat ants are diurnal (Greenaway 1981) and more generally might limit their activity in low light or as a function of time of day, independently of temperature. Furthermore, meat ants are vulnerable to desiccation, which could be exacerbated in windy conditions.

Statistical analyses were conducted in several planned stages and applied separately for foraging and defensive swarms. All analyses were applied using R version 4.2.2 (R Core Team 2023).

The first set of analyses included all potential predictors of foraging or swarm activity to gauge the effects of ground surface temperature, light, wind speed and time of day. Generalized additive models were applied using the “mgcv” package version 1.8-38 (Wood 2011). Smooth terms were included for all continuous variables to allow for non-linear effects. Nest size (number of entrance holes) and nest ID were also included as additional factors or as a random effect, respectively. This was because the number of workers moving along trails or the intensity of the swarm across the nest can be expected to be positively correlated to the size of the colony (inferred from the number of entrance holes), while also dependent on the unknown density of aphids in the destination tree or the temperament of a colony (eg see Pinter-Wollman et al. 2012), variables specific to a given nest (so captured by nest ID).

In the second set of analyses, generalized additive models focused on predictor variables that were identified as contributing statistical effects that were clearly distinguished from zero. These are labeled in later sections as “adjusted” temperature-dependent performance curves because they include the additional effects of these variables alongside temperature. To provide clearer resolution of the dominating effect of temperature for later analyses (see “Computing available hours for foraging and defence”), additional models were applied to foraging and swarm activity that only included surface temperature as the primary variable of interest. These models are labeled in later sections as “primary” temperature-dependent performance curves.

Finally, the emergence of ants in defensive swarms from the nest was examined using a Bayesian change-point random regression model using the ‘mcp’ package version 0.3.0 (Lindeløv 2020). This analysis focused on estimating emergence at the lower temperature threshold of the swarm performance curve. This was because there were too few data points to also estimate the upper temperature threshold associated with the curve (ie on the day nests were studied in summer, nest surface temperatures evidently did not typically exceed the upper temperature tolerance of workers). For the upper temperature threshold, we relied on the maximum temperature that workers were observed to still emerge from a nest, 47.7 °C. This is likely a liberal estimate of the upper temperature threshold, with a conservative estimate converging on 42.2 °C (the median non-emergence temperature, range 37.3 to 47.4 °C, *N*_observations, nests_ = 10, 3).

### Measuring nest surface temperatures

We selected six of the eleven nests used to estimate foraging and defensive swarm performance curves (previous section) for long-term monitoring based on their spatial position in the environment and their subsequent regime of sun exposure and shading. For these six nests, either a combined temperature and relative humidity probe or thermistor probe (temperature only; PB-4720) attached to a Tinytag Plus 2 data logger (TGP-4020/TGP-4505) was positioned over the approximate center of the nest. Data was collected at 30-min intervals for at least two years (probes were initially placed on nests on 5 November 2018 and removed 14 March 2021). Occasional gaps in the data occurred because of battery failure (rare) or the probe becoming displaced by kangaroo, wombat or bird activity.

To determine whether nests differed in surface temperature, data was first parsed to daytime temperatures only and then modeled using three separate splines: no random effect for nest ID, random intercept for nest ID, random intercept and slope for nest ID. Splines were applied using the “gamm4” package version 0.2-6 (Wood 2020). Bayesian Information Criterion (BIC) was used to identify the relative support for the inclusion of nest ID random effects, which was further confirmed using a restricted likelihood ratio test implemented with the “RLRsim” package version 3.1-8 (Scheipl et al. 2008) using 10,000 simulated values. Two sets of analyses were applied. First, we established that all nests began from the same starting temperature each day by confirming the minimum daily temperature of nests did not vary statistically among the six focal nests. This reflected that all nests ultimately cooled to the same surface temperature overnight. Second, we examined the range in daily surface temperatures, with any deviations among nests indicating that nest location produced differences in nest surface temperature. Other comparisons of median and maximum daily surface temperatures were also considered but highlighted the same qualitative differences among nests.

### Computing available hours for foraging and defence

The number of daily hours available for foraging and defensive swarm activity was computed by combining emergence and optimal activity windows estimated from temperature-dependent performance curves, with the number of hours a nest experienced temperatures within that window. Emergence temperatures reflected the estimated lower and upper temperature thresholds that workers were observed to move along tree trails or exit the nest to swarm. Optimal temperatures reflected the more restricted temperature window in which the number of workers moving along tree trails or the speed of swarms was at least 90% of maximum capacity (corresponding to the apex of the performance curve). Taken together, inferences drawn from emergence and optimal temperatures provided two contrasting estimates of foraging and defence capacity.

The number of 30 min intervals between the lower and upper temperature thresholds were summed over the day to compute an inferred number of hours available for foraging or defending the nest against predation. Splines were applied in a similar fashion as described in the previous section and were used to establish the extent to which the number of available hours for foraging and nest defence differed among the six focal nests. This was done using the “gamm4” package to apply models with no random effect for nest ID, random intercept for nest ID, random intercept and slope for nest ID. A restricted likelihood ratio test was then applied to these models using the “RLRsim” package and 10,000 simulations to identify whether random effects contributed statistical effects to the model.

### Determining whether nests compensate for restrictions on activity

Colonies might offset temperature-dependent restrictions on the hours available for foraging by simply increasing foraging effort. In this case, daily foraging hours would not be predictive of nest size and specifically because nests positioned in locations with more restrictive temperature regimes compensated by establishing more foraging and tree trails. We used trail number as an index of foraging effort because long-term study of colonies in this population has shown nests with more foraging and tree trails are larger, have higher annual growth and remain active for more years than nests with fewer trails (Ord 2023). This suggests the number of trails established by a nest plays a critical supporting role through its effect on nest foraging capacity. The average number of entrance holes calculated across all years a nest had been monitored (either 10 or 11 years) was used to estimate nest size (see Supporting Information). The number of foraging and tree trails established by each nest was first standardized by computing a random effects regression model in which trail number was modeled as a function of nest size, with a random intercept and slope for nest ID. This analysis leveraged data from all nests in the population (*N*_nests_ = 111), for all years surveyed (2015 to 2024), and was applied using the “lme4” package version 1.1-28 (Bates et al. 2015). The total number of foraging and tree trails observed for each nest in each year was log10-transformed to improve model fit. Coefficients for individual nests were extracted from the main model (Table S1) and those estimates for the six focal nests back-transformed to provide the number of foraging and tree trails established by these nests, averaged across years, and independent of nest size. This standardized estimate using data from multiple years controls for momentary fluctuations in trail number that might occur because of daily changes in available food resources. We then examined nest size and the number of foraging and tree trails independent of nest size—an index of foraging effort—as a function of the inferred available foraging hours computed for each nest. If nests compensate for temperature-dependent restrictions on foraging time, nest size should not vary as a function of available foraging hours, while the number of foraging and tree trails established by a nest should increase as the number of available foraging hours decreases across nests.

### Evaluating long-term colony growth

Given colonies did not seem to offset restrictions on foraging time through increasing foraging effort (see previous section and ‘Results’), we examined longitudinal changes in nest size across years to determine the consequences of nest location—and its subsequent influence on the temperature conditions experienced at the nest—on colony growth. Three basic models were applied to each nest: an intercept only model that assumed no annual change in nest size, a linear model that assumed a progressive change in nest size across years, and a quadratic model that assumed a non-linear change in nest size across years. Akaike Information Criteria with a correction for sample size (AIC_c_) was used to compare the level of support for each model. The best supported model was indicated by the lowest AIC_c_ value, although any other model within two units of this model was also considered to be well supported. Effect sizes in the form of *t*-values were used to evaluate the magnitude and direction of changes in nest size across years.

## Results

### Temperature-dependent performance curves

Exploratory analyses that considered all potential determinants of foraging and swarm activity (temperature, light, wind, time of day and nest size) identified surface temperature as the variable with the largest effect on activity, followed by nest size and time of day (Tables S2-S3). Comparison of the primary performance curves (that modeled only the influence of surface temperature) with the adjusted performance curves (that included the additional effects of time of day and nest size) showed negligible differences in the shape of the computed curves associated with temperature. Effective Degrees of Freedom (EDF), which reflects the curvature of the computed trend line (a linear trend would be EDF = 1), were largely comparable between primary and adjusted curves for temperature (eg Table 1a—EDF_surface temperature_ of worker presence on trails, primary vs adjusted: 3.93 vs 3.87; worker number on trails: 3.46 vs 3.35; Table 1b—EDF_nest temperature_: 3.50 vs 3.49). Visual inspection of plotted performance curves for foraging and swarm activity further confirmed this similarity between primary and adjusted curves (Figure S1). Peak foraging activity occurred at 29.7 versus 31.9 °C, with an optimal window (90% from maximum capacity) of 26.6 to 33.2 °C versus 28.9 to 34.8 °C for primary versus adjusted curves, respectively (the adjusted curve was benchmarked to the median nest size and noon). Peak swarm activity occurred at 35.1 versus 35.7 °C, with an optimal window of 23.5 to 42.3 °C versus 26.2 to 42.9 °C, respectively. In general, modeling activity exclusively as a function of surface temperature provided an adequate means of estimating foraging and defensive swarm performance curves (NB: data for light, wind, time of day and nest size are shown in Figure S2 with summary statistics presented in Table S4).

**Table 1.** Generalised additive models used to estimate performance curves of (a) foraging and (b) swarm activity. Foraging activity was estimated using a hurdle model that combined a logistic model of worker presence on trails and a Poisson model of the number of workers once present. Swarming activity was focused on instances where ants were recorded on the surface of the nest, with the lower and upper tolerated temperatures of examined separately (see Table S6). Primary activity curves considered only the effect of surface temperature, while adjusted activity curves included additional predicts of nest size and time of day.

a. foraging activity			
primary curve			
Parametric coefficients	b	z	p
intercept (presence on trail)	1.75	6.14	< 0.001
intercept (number on trail)	−0.66	−2.06	0.04
Smoothing term (k = 5)	EDF	*c*2	*p*
surface temperature (presence on trail)	3.93	701.46	< 0.001
surface temperature (number on trail)	3.46	68.66	< 0.001
nest ID (presence on trail)	9.91	1591.8	< 0.001
nest ID (number on trail)	7.86	25.59	< 0.001
deviance explained = 59.5%, *N*_nests, observations_ = 11, 237			
adjusted curve			
Parametric coefficients	*b*	*z*	*p*
intercept (presence on trail)	0.62	1.25	0.21
intercept (number on trail)	−1.89	−3.78	< 0.001
nest size (presence on trail)	0.04	2.99	0.003
nest size (number on trail)	0.04	2.88	0.004
Smoothing terms (k = 5)	EDF	*c*2	*p*
surface temperature (presence on trail)	3.87	469.59	< 0.001
surface temperature (number on trail)	3.36	63.35	< 0.001
time of day (presence on trail)	3.97	345.66	< 0.001
time of day (number on trail)	3.00	10.08	0.02
nest ID (presence on trail)	8.87	784.05	< 0.001
nest ID (number on trail)	4.91	9.68	0.04
deviance explained = 67.3%, *N*_nests, observations_ = 11, 237			
b. swarm activity			
primary curve			
Parametric coefficients	*b*	*t*	*p*
intercept	12.74	39.96	< 0.001
Smoothing term (k = 5)	EDF	*F*	*p*
nest surface temperature	3.50	30.87	< 0.001
adjusted *r*^2^ = 0.29, *N*_nests, observations_ = 11, 148			
adjusted curve			
Parametric coefficients	*b*	*t*	*p*
intercept	11.27	17.62	< 0.001
nest size	0.05	2.51	0.01
Smoothing terms (k = 5)	EDF	*F*	*p*
nest surface temperature	3.49	37.67	< 0.001
time of day	2.97	10.81	< 0.001
adjusted *r*^2^ = 0.44, *N*_nests, observations_ = 11, 148			

Based on the primary performance curve, workers were observed moving along tree trails, on average, between 15.9 and 43.1 °C (Fig. 1b). The temperature window for defensive swarms was a little wider, ranging from 13.1 to approximately 47.7 °C (Table S5a; Fig. 1c). Individual nests varied in their computed emergence temperatures for defensive swarms (Table S5b), but sampling was limited to a single day in summer and winter for each nest and individual nest estimates have therefore been estimated with low statistical power.

### Nest surface temperatures across years

The minimum daily surface temperature of nests, which corresponded to those at dawn, were statistically indistinguishable among the six focal nests. The best supported model of daily variation in minimum surface temperature had no random effect for nest ID, which was also the model that provided the best fit to the data (Table 2). This reflects nests cooling overnight to a common surface temperature resulting in a virtually identical starting temperature across nests each morning (eg see Figure S3). Over the course of the day, however, nest location clearly had an impact on nest warming, with the magnitude of nest temperature ranging widely among nests. A model with a random intercept for nest ID was the best supported model and provided the best fit to daily fluctuations in the range of temperatures experienced by each nest (Table 2). The differences in surface temperatures among nests was especially obvious in winter, with the two most extreme nests—the exposed nest of XI and the sheltered nest of LVI—experiencing winter daily maximums of 25 to 30 °C and 15 to 20 °C, respectively (see Figure S3).

**Table 2.** Splines used to model longitudinal daily fluctuations in nest surface temperature (see Figure S4b). Differences in temperatures among nests were evaluated by fitting alternative models with and without random effects for nest ID and applying restricted likelihood ratio tests (RLRT) to compare the fit of those models.

model	BIC	ΔBIC	RLRT	*p*
minimum surface temperature				
no random effects	16261.7	0.0		
random intercept	16266.2	4.5		
random intercept + slope	16273.2	11.5		
RLRT: random intercept + slope vs intercept only			1.00	0.11
RLRT: random intercept vs no random effect			3.51	0.02
range surface temperature				
no random effects	20008.9	64.0		
random intercept	19944.8	0.0		
random intercept + slope	19953.4	8.5		
RLRT: random intercept + slope vs intercept only			0.0	1.00
RLRT: random intercept vs no random effect			72.06	< 0.001

### Available hours for foraging and defence

**Figure 2.**
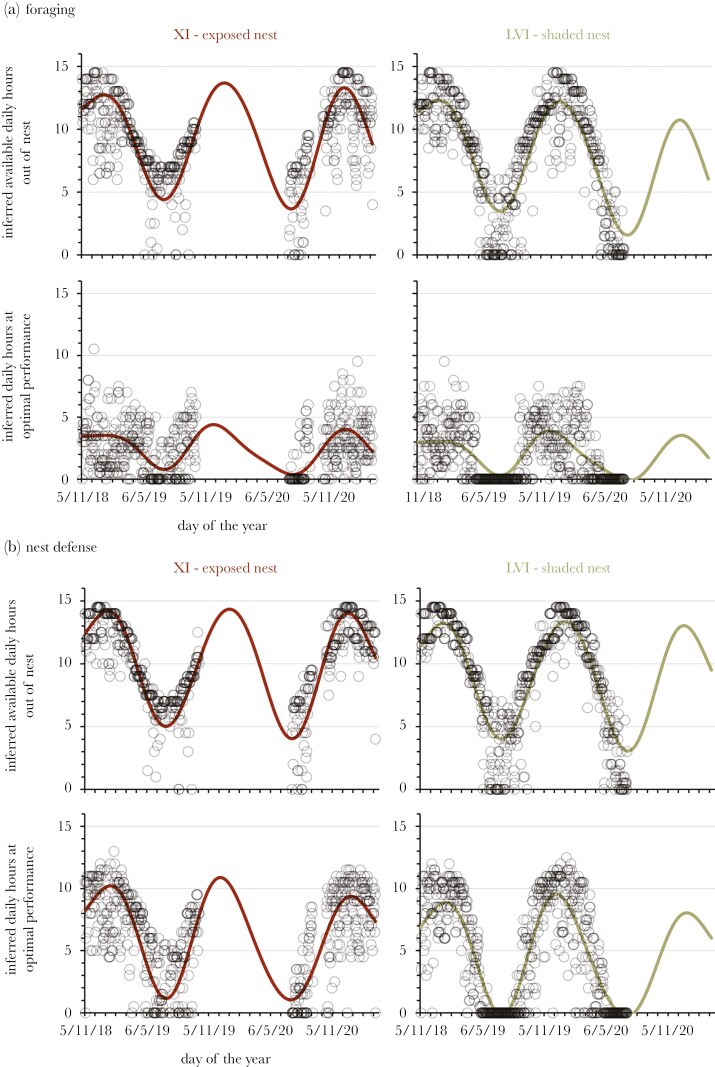
Nest XI and LVI were positioned in locations that received the most solar exposure and shading, respectively. These nests recorded the largest differences in nest surface temperatures (Figure S3) and subsequently differed the most in available hours for (a) foraging and (b) nest defence. The top rows in (a) and (b) show the time available based on the lower and upper temperature thresholds tolerated by ants (the minimum and maximum temperatures of activity shown in Fig. 1b, c), while the bottom rows are times available at optimal performance, defined as the window of temperatures corresponding to at least 90% of the maximum number of workers observed moving along tree trails or the maximum speed of swarms (dashed lines in Fig. 1b, c). Open circles are the number of inferred daily hours for activity. Trend lines are splines computed by the models reported in Table S6. Gaps in the data occurred because of battery failure or probes being displaced by kangaroos, wombats or birds.

**Figure 3.**
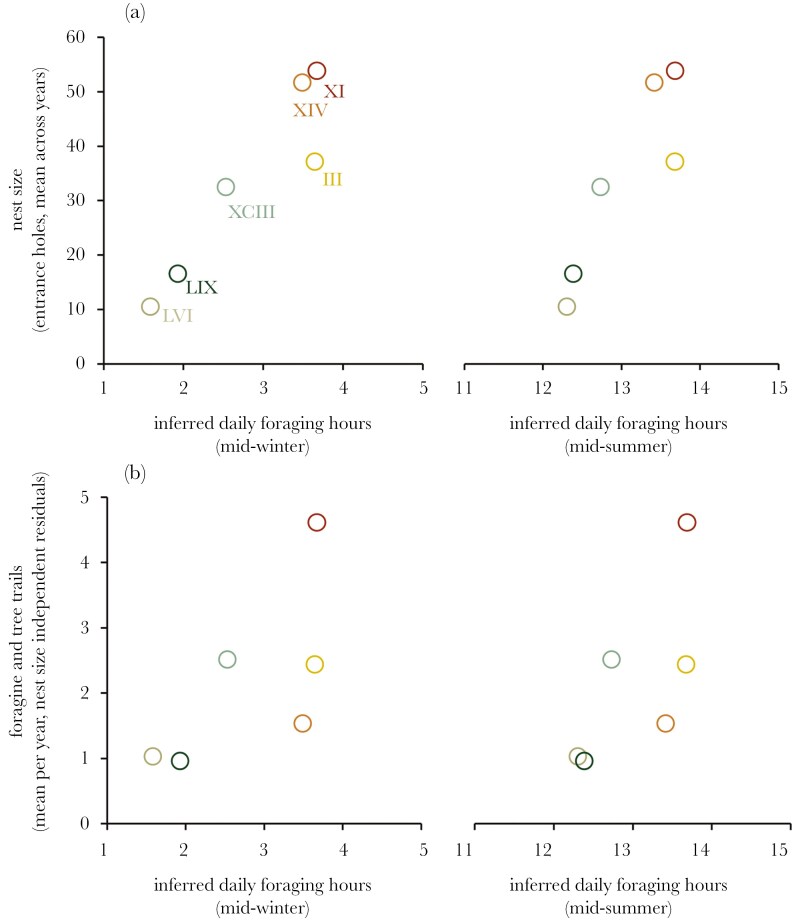
(a) Nest size and (b) the number of feeding trails established by nests (independent of nest size) indexing foraging effort, as a function of time available for foraging in mid-winter and mid-summer (the base and apex of computed trends in foraging hours shown in Figure S4a). If colonies compensated for poorly positioned nests and subsequent temperature constraints on activity, there should be no discernible trend between nest size and foraging time, and a negative trend between the number of trails maintained by a nest and the hours available for foraging.

The inferred time available to forage or provide nest defence (via swarms) varied widely among the six focal nests, irrespective of whether estimates were based on the lower and upper temperature thresholds or optimal temperature window for activity. All models had at least a random intercept for nest ID, and in the case of foraging activity between the lower-upper temperature threshold, a random slope for nest ID (Table S6). The rank of each nest mirrored those of their nest surface temperatures (see previous section and Figure S4). Specifically, the two nests with the most extreme temperature microclimates—XI and LVI (Fig. 2)—showed large differences in the number of available hours for activity, with the remaining nests distributed between these two extremes (Fig. 3). The differences between nests were again especially obvious in winter, with the two most exposed nests (XI and XIV) having an average of 3.5 to 4 h available for foraging on an average winter’s day, compared to the two most sheltered nests (LVI and LIX) having less than half that (< 2 h; Fig. 3). There were also many days in winter where the more sheltered nests likely had no opportunity to forage or defend the nest from predation (Figure S4-S5). That is, nests positioned in locations getting more shade had less time to perform key behaviors, especially in winter.

Nevertheless, nests that receive more sun were still prone to restricts on activity. While the on-average time for foraging on a typical summer’s day was higher for the most exposed nest XI compared to the most sheltered nest LVI (13.7 h versus 12.3 h, respectively; Fig. 3), hot summer days resulted in more restrictions on activity for the exposed nest XI (compare estimated daily foraging hours below the trend line in summer between XI and LVI in Figs 2 and S4). That is, there is a compromise being made by a colony in placing its nest in an exposed location (away from the shade of the tree line) because surface temperatures will routinely exceed the upper threshold for workers on hot summer days.

### Activity hours and foraging effort

A robust positive trend was evident between inferred foraging hours and nest size (Fig. 3a). This implies nest size was detrimentally impacted by temperature constrains on the capacity of a nest to forage. Nests with the least available foraging hours were also those that established the fewest number of foraging and tree trails, independent of nest size (Fig. 3b). This shows that colonies were unable to compensate for fewer foraging hours by increasing foraging effort in those periods, and this has impacted the size of nests.

### Long-term nest growth

**Figure 4.**
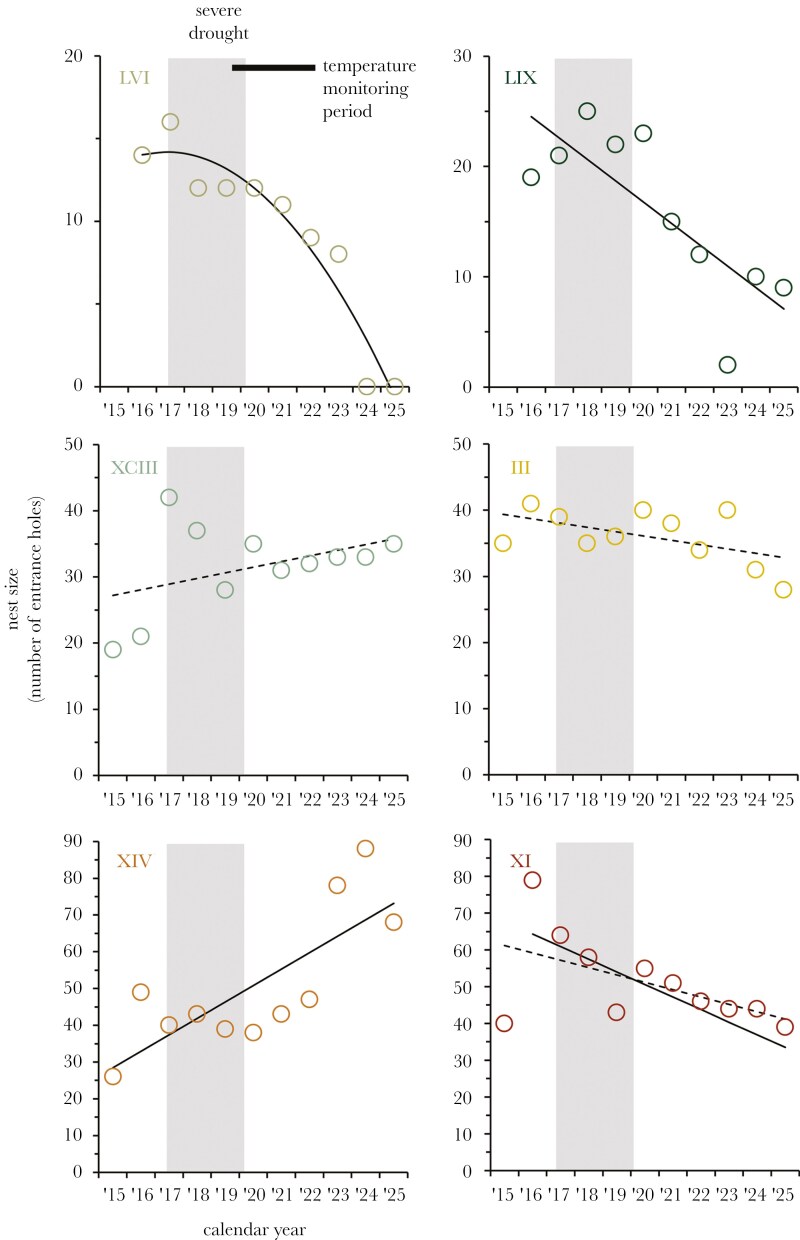
Nest growth measured by changes in nest size across years. Panels are ordered from top-down left to right from the most shaded nest (LVI) to the most exposed nest (XI). Trend lines are those models identified in Table 3 to have large statistical effects (*t* > 2; solid lines) or credible levels of support (ΔAIC_c_ ≤ 2.0; dash lines). The gray corresponds to years of severe drought (the worst in recorded history for Australia; data reported in Ord 2023). The period over which nest surface temperatures were recorded (Figure S3b) is shown by the black bar. Nests LVI and LIX were not surveyed in 2015.

Annual changes in nest size varied widely among the six focal nests, and in a manner suggesting nest position has had a long-term impact on nest growth. Nests with the most restrictions on activity because of shading—nests LVI and LIX—have experienced dramatic declines in nest size (Table 3; Fig. 4). So much so, the worst effected nest was extinct by the end of the study in 2024 (nest LVI). Other nests with more balanced temperature microclimates have either maintained their size or tended to increase in size over the years. The exception has been nest XI, the most exposed nest of the six, which seems to have experienced a general decline in size (Table 3; Fig. 4).

**Table 3.** The extent to which the size of nests (number of entrance holes) varied across years, assuming no change (intercept only model), linear change or quadratic change in nest size across years (see Fig. 4).

model	AIC_c_	ΔAIC_c_	*t*_years_	*t*_years_^2^
LVI, *N*_years monitored_ = 10				
no annual change (intercept only)	66.9	14.5		
linear annual change	54.2	1.8	−5.98	
quadratic annual change	52.4	0.0	0.53	−2.87
LIX, *N*_years monitored_ = 10				
no annual change (intercept only)	73.2	5.5		
linear annual change	67.7	0.0	−3.64	
quadratic annual change	72.6	4.9		
XCIII, *N*_years monitored_ = 11				
no annual change (intercept only)	77.5	0.0		
linear annual change	79.3	1.8	1.40	
quadratic annual change	82.4	4.9		
XIV, *N*_years monitored_ = 11				
no annual change (intercept only)	100.4	6.5		
linear annual change	93.9	0.0	3.77	
quadratic annual change	97.0	3.1		
III, *N*_years monitored_ = 11				
no annual change (intercept only)	66.5	0.0		
linear annual change	66.7	0.2	−1.90	
quadratic annual change	67.6	1.1		
XI, *N*_years monitored_ = 11				
no annual change (intercept only)	90.6	0.0		
linear annual change	90.5	0.0	−1.97	
quadratic annual change	95.2	4.7		
2015 removed (*N*_years monitored_ = 10)				
no annual change (intercept only)	90.6	16.2		
linear annual change	74.3	0.0	−4.61	
quadratic annual change	75.9	1.6	−3.35	1.97

Drought seems to account for additional fluctuations in nest size, with all nests suffering a general decline or stagnation in nest growth from 2017 to 2019. This period corresponded with the onset of the worse drought in Australia’s recorded history, which reduced the annual rainfall recorded at this site from a 19-year average of 646 ml to an unprecedented low of 314 ml in 2019 (Ord 2023). For those nests positioned in locations that were documented to be at the lower or upper ranges of nest surface temperatures—shaded nests LVI and LIX and exposed nest XI—drought might have been the initial trigger of nest decline, from which these nests have been unable to recover. In contrast, the remaining nests have either remained at their pre-drought nest size or begun to increase in size post-drought.

## Discussion

Central place foragers that invest heavily in the construction of a central refuge benefit from having a shelter between bouts of foraging that protects against predation and daily or seasonal fluctuations in environmental conditions. The cost of construction and maintenance of this central place is subsequently offset by improved survival and reproduction. This assumes the central refuge is positioned appropriately for exploiting available food sources in the surrounding environment and that the environment itself remains broadly stable over time. The best sites for refuges (eg nests) are often the subject of competition (Hubbell and Johnson 1977; Bengston and Dornhaus 2015; Ord 2023), which means the ideal placement of a central refuge is not always achieved. The surrounding environment can also be detrimentally impacted by a range of natural events, including drought (Couper et al. 2021), which are increasing in frequency and severity with the climate crisis (Karl and Trenberth 2003; Rahmstorf and Coumou 2011; Coumou and Rahmstorf 2012; Screen and Simmonds 2014; Jain et al. 2022). The constraint on mobility imposed by occupying a single refuge has the potential to make some central place foragers vulnerable to prolonged or severe changes in environmental conditions.

Meat ants fit the classic description of a central place forager (Harkness and Maroudas 1985; Houston and McNamara 1985). These ants are iconic features of the Australian landscape because of the construction of large, conspicuous ground nests—a colony’s central refuge—and often dominant environments in many parts of south-eastern Australia (Anderson and Patel 1994; Anderson and Majer 2004). The extensive geographic range of meat ants (covering ~30% of the continent; www.ala.org.au) implies this species accommodates a diverse range of conditions. For similar reasons, ants in general are often described as poor bioindicators of ecosystem health because of their putative resilience to environmental stress (Andersen 1997; Whitford et al. 1999; Philpott et al. 2009). Meat ants might therefore be considered a “hardy” central place forager and have a reasonable capacity to cope with adverse conditions (Parr and Bishop 2022). Data from previous study (Ord 2023) suggests meat ant colonies attempt to position nests to maintain the nest within a preferred temperature window. The current study shows nest placement has a direct impact on a colony’s capacity to forage and defend itself from predation. The thermal niche of a given nest location is defined by the surrounding vegetation and general topography of the area, which determines the balance of warming from direct sun and cooling from shade. Our data shows that getting the placement of a nest wrong has a tangible impact on the growth and survival of the nest (Fig. 4) and colonies appear not to be able to compensate through changes in behavior (Fig. 3).

Competition among colonies is an important factor determining the spatial distribution of meat ant nests at this site, with a pattern of repulsion evident in the spacing of large nests relative to each other (Ord 2023). The best predictor of nest growth and survival are the number of foraging and tree trails maintained by a nest (Ord 2023), suggesting food is a critical resource and likely the focus of competition among colonies (see also Han and Elgar 2020). The nest effectively anchors a colony in place for many years, and a large, established colony prohibits other colonies constructing a nest within a competitive exclusion zone (eg within ~100 m; see Ord 2023). More broadly, there is a narrow habitable zone for meat ant colonies at this site that tracks the woodland-grassland boundary (Fig. 1a). Our data now defines this zone by the thermal tolerance of meat ants and the surface temperatures that workers are able to remain active outside of the nest (Fig. 1b, c). Competition likely accounts for some colonies placing nests in locations closer to the periphery of the habitable zone (eg nests LVI, LIX and XI), and why the temperature conditions at the surface of the nest (Table 2; Figure S3) and subsequent capacity for activity (Table S6; Fig. 2, S4 and S5) varied among the nests monitored.

Nevertheless, all nests in this study were explicitly selected because they were established nests at the outset (eg at least three have been active since 2010: XCIII, LIX and III; Ord 2023). Prior to the onset of drought in 2017, most focal nests seemed to have been experiencing some level of growth (Fig. 4). Nests positioned closer to the edges of the habitable zone—those subject to more shade (LVI and LIX) or more sun exposure (XI)—did not to seem have been adversely impacted by any temperature-dependent restrictions on activity (Fig. 4). We suspect this was because food resources were not limiting in pre-drought years. During drought, however, most colonies across the population became stressed and to such an extent an unprecedent level of nest budding was observed as colonies in this population attempted to shift closer to the woodland-grassland boundary or into the woodland itself (Ord 2023). There was almost certainly a reduction in food resources during this period (eg see Gibb et al. 2019), and this appears to have triggered the decline of the sheltered nests LVI and LIX (Fig. 4), which exhibited the tightest restrictions on activity (Figs 2, S4, and S5). The reasons for the decline of nest XI, the most exposed nest, were less unclear. This nest had the most available hours, on-average, for workers to be active outside of the nest (Figs 2 and 3). However, it seems this nest’s exposure on hot summer days during the worst of the drought compounded the impact of food limitation. None of these three periphery nests have recovered in the years post-drought, and the worse effected colony was extinct before the end of the study (LVI). None of these nests were predated during the study and none were colonies that attempted to shift location during the drought through nest budding (TJO , unpublished data). Nest decline therefore cannot be attributed to predation or the transfer of the colony to a new nest.

The temperature tolerances that we recorded in this meat ant population were comparable to those previously documented by other studies on this species, both in the field (Mobbs et al. 1978; Greenaway 1981; Andrew et al. 2013) and lab (Hemmings and Andrew 2017). This implies little plasticity or population divergence in thermal performance windows for this species. Our data shows meat ants do not seem to compensate for temperature-dependent restrictions on activity from poor nest placement or changes in the conditions impacting the microclimate of nests (contrast with other ectotherms: Moss et al. 2023; and plasticity in foraging effort in other ants: Barbee and Pinter-Wollman 2023). Given meat ant colonies are long-lived (data reported in Ord 2023) and rarely establish new nests (data reported in Greaves and Hughes 1974; Greenslade 1975; Ord 2023), the long-term occupancy of nests makes this central place forager especially susceptible to unprecedented environmental change and at a heightened risk of local extinction. Meat ants often dominant the invertebrate fauna in many south-eastern Australian environments (Stevens et al. 2002; Gibb and Hochuli 2004; Palfi et al. 2017;  Gibb et al. 2019), and this is certainly the case at this site as well (TJO, unpublished data). The specific contribution of this species to ecosystem functions is hard to gauge beyond their general dominance of a key function group (ants, and invertebrates more generally; Folgarait 1998; Gibb and Hochuli 2003; Andersen et al. 2009; del Toro et al. 2012; Andersen 2018). Meat ants have been implicated in seed dispersal and resource transfer within environments (Gibb 2003; Bebawi and Campbell 2004; Palfi et al. 2017). They have the potential to be important ecosystem engineers through the construction of their large ground nests as well (eg Sanders and van Veen 2011; De Almeida et al. 2020a, 2020b; Zhong et al. 2021). The decline of meat ants at a locality therefore has some potential to translate into broader consequences for the environment as well.

What is clear, however, is that some central place foragers will likely be especially vulnerable to prolonged, adverse changes in environmental conditions. Our data shows this to be certainly the case for a terrestrial species that is arguably an ecologically dominant and important influence on its local environment. Meat ants could also be argued to be a central place forager that should be quite resilient to disturbance, yet even this species was sensitive to long-term changes in temperature microclimates and resource availability, and in circumstances (drought) that are likely to become increasingly more frequent in the future. Other central place foragers are likely to be similarly exposed to prolonged environmental change (Fagan et al. 2007; Pichegru et al. 2010; Boyd et al. 2014; Wakefield et al. 2014; Sagata and Gibb 2016; Brewster et al. 2021; Gicquel et al. 2022; Kendall et al. 2022; McHuron et al. 2023), and not just those that invest heavily in the construction of a central refuge. For example, resource depletion halos around central refuges used by coral reef fish (Madin et al. 2022) and other central place foragers (birds: Weber et al. 2021; lizards: Chase 1998) implies any major disruption to resource availability around a central place could have detrimental impacts on the capacity of a central place forager to support itself (Pichegru et al. 2010; Weber et al. 2021). Factors that impact the density of vegetation and subsequent shading in environments (eg fire suppression that results in shrub encroachment) can have profound effects on the capacity of ectotherms to be active (Brewster et al. 2021; see also Moss et al. 2023). Temperature-dependent constrains on activity have similarly been used to predict the widespread extinction of many lizard species under climate change (Sinervo et al. 2010). Unless species compensate for environmental change by changing their foraging behavior (eg Weber et al. 2021) or moving their central place to a new area outside of the zone of impact (eg Pinter-Wollman and Brown 2015), central place foragers are potentially faced with a bleak future of a heightened risk of local extinction. Even for the putative stress tolerant meat ant, there appears to be little recourse for a poorly situated nest and limited capacity for long-lived colonies to shift this central refuge to a more favorable location.

## Supplementary material

Supplementary material is available at *Behavioral Ecology* online.

araf062_suppl_Supplementary_Tables_S1-S6_Figures_S1-S5

## Data Availability

Analyses reported in this article can be reproduced using the data provided by Ord and Blazek (2025).
