# Supplementary material for: Central place foraging in an ectotherm and the long-term liability of selecting the “wrong” central place
Source: Behav Ecol. 2025 May 29;36(4):araf062. doi: 10.1093/beheco/araf062 (PMC12187638; doi:10.1093/beheco/araf062)
Supplement: araf062_suppl_Supplementary_Tables_S1-S6_Figures_S1-S5 [file araf062_suppl_supplementary_tables_s1-s6_figures_s1-s5.docx]

Supporting Information for ‘Central place foraging in an ectotherm and the long-term liability of selecting the ‘wrong’ central place’

*Study system*

The population of meat ants is found on a private property consisting of cleared pasture previously used for low intensity cattle farming until 2007. This grassland transitions abruptly to remnant dry sclerophyll eucalyptus woodland. Meat ant colonies tend to place their nests within approximately 50 metres of this grassland-woodland boundary (Figure 1a; Ord 2023). Nests range in size from just a few entrance holes to over a hundred, which corresponds to approximately 10,000 to over a million ants, respectively (the number of entrance holes is tightly correlated to the number of resident ants (*r*^2^=0.95) based on seven excavated nests reported by Greaves and Hughes 1974; see Ord 2023 for formal calculation of regression analysis of these data). Each nest hole leads to an independent set of galleries (Ettershank 1968; Greenslade 1973), and when abandoned, the entrance becomes quickly filled with debris and the hole becomes invisible from the nest surface in a matter of days (XXX personal observation). Taken together, the number of visible entrance holes provides an accurate means of estimating the size of the living colony within the nest. Colonies typically occupy a single nest (median number of connected nests = 0; XXX unpublished data), although massive colonies can distribute themselves across as many as eight nests connected by cleared trails.

*Estimating temperature-dependent performance curves*

Tree trails were selected for quantifying foraging activity for two reasons. First, tree trails could be readily identified prior to any activity starting because trails were maintained by the colony through regular clearing. Second, tree trails were often consistently used from one day to the next, so activity was predictable. Foraging trails, in contrast, were generally temporary, shifting in location from one day to the next, and often only identifiable once a steady flow of workers from the nest began. Regardless, the traffic of workers along either tree or foraging trails can be expected to provide a comparable measure of worker activity for a given nest, meaning the focus on tree trails specifically was incidental to the objectives of the study. Worker counts along tree trails were made over 1 minute from digital videos recorded using a camcorder fixed to a tripod, positioned approximately 50 cm above the trail and roughly halfway between the nest and the destination tree.

Defensive swarm activity was measured using digital videos recorded using a camcorder positioned on a tripod 1.5-2 metres above the surface of the nest so that the entire surface of the nest was in view. The swarm was recorded for approximately 3 minutes, with the first 20 seconds following the nest scrape used to quantify the movement speed of ants as they swarmed across the surface of the nest. This was done using the Matlab-based program ‘Analysis of Image Movement’ (Peters et al. 2002). Estimates of swarm speed were calibrated to millimetres moved per second using a notebook of known size that was placed in frame during video recordings.

Trail and nest surface temperature was measured using an Extech Infra-red digital surface thermometer gun. Downwelling light was measured using a LI-205A handheld light meter connected to a LI-190SA Quantum Sensor, with the senor pointing directly up to the sky. Time of day was noted at the start of video recording. Wind speed was averaged over 10 seconds using a WeatherMate hand-held thermo-windmeter (WM20) positioned approximately 1-2 cm above the substrate of the trail or nest.

For the first (all inclusive) set of analyses for foraging activity, a generalized additive model with a binomial error distribution was applied using the ‘mgcv’ package. Foraging activity was converted to a score of 0 (no workers observed on the trail) or 1 (at least 1 worker observed on the trail) to provide a first pass evaluation of factors associated with the presence of workers on tree trails. For swarm activity, a generalized additive mixed model was applied with a gaussian error distribution using the ‘mgcv’ package. In this model, nest ID was included as a random intercept and slope, and focussed only on observations when ants were seen swarming on the surface of the nest (i.e., non-emergence observations—swarm speed = 0—were excluded).

In the second (focussed) set of analyses, foraging activity on tree trails was modelled using the ‘mgcv’ package with a zero inflated Poisson generalized additive model applied with a ‘ziplss’ error distribution (known as a zero inflated (hurdle) Poisson location-scale model). This hurdle model combines a logistic model to estimate the presence/absence of workers on tree trails and a Poisson model to estimate the number of workers moving along the tree trail once present. In both parts of the model, worker activity was modelled as a function of trail surface temperature, time of day and nest size, with smoothing terms for the non-linear effects of temperature and time of day based on 5 knots. This number of knots was determined from exploratory analysis that indicated this value provided a reasonable visual approximation of the data. Swarm activity was again only focussed on observations when ants were swarming on the surface of the nest, with emergence from and retreat back into the nest examined separately (next paragraph). This model used a generalized additive mixed model applied with a gaussian error distribution using the ‘mgcv’ package, with nest ID included as a random intercept only (to be consistent with the foraging model). Nest size was included as a linear factor, while nest surface temperature and time of day were modelled with smoothing terms based on 5 knots.

To examine the lower-temperature emergence of ants in defensive swarms, the data was first parsed to exclude swarms occurring above 35^o^C, which was the approximate peak of the swarm performance curve (from analyses described in previous paragraph). A regression model with a random intercept for nest ID was then applied using a Bayesian change-point random regression model with the ‘mcp’ package, which specified a flat slope of swarm speed across temperatures prior to the change point (reflecting no emergence from the nest over those temperatures), with a level jump in swarm speed at the change point, followed by a linear increase in swarm speed as nest surface temperature increased towards 35^o^C. The parameter of interest was specifically the temperature at the change point, not the slope (or its shape) after the change point. Other models with non-linear changes in swarm speed following the change point and inclusive of all data (i.e., across the full range of temperatures studied; -6.8^o^C to 47.7^o^), resulted in a poorer estimate of the change point temperature and were not considered further.

*Measuring nest surface temperatures*

The probe of data loggers was housed within a custom-made length of PVC pipe (~10 cm) to prevent the metal casing of the probe heating from direct sun exposure and distorting temperature measures. The PVC pipe was secured with four legs of fencing wire, which were used to anchor the probe in place on the nest surface and at a distance of approximately 0.5-1 cm above the surface of the nest. This distance was a rough approximation of the position of a worker standing on the surface of the nest.

Prior to statistical analysis, temperatures between sunset and sunrise were excluded using daily times taken from Geoscience Australia’s ‘geodetic calculator’ (https://geodesyapps.ga.gov.au/sunrise) for GPS coordinates at the centre of the field site (S32° 29.544' E149° 58.583') and over the dates probes were deployed on nests, relative to Australian Eastern Standard Time.

*Computing available hours for foraging and defence*

A conversion equation was formulated to transform temperature thresholds taken from performance curves to equivalent values of nest surface temperature recorded from permanently placed probes. This was required because temperatures taken using the infra-red thermometer gun were those recorded of the substrate surface, whereas those from probes were effectively ambient air temperatures recorded immediately above that surface. A backward elimination process of fractional polynomials was applied using the ‘mfp’ package version 1.5.2.2 (Heinze et al. 2022) to identify the most appropriate transformation, which was then applied with an additional factor distinguishing the type of probe used (temperature only vs combined temperature and relative humidity probe: Table S7; Figure S6). The additional factor was included because the design of the two probes was different and proved to be influential in determining subtle nuances in the recorded temperatures.

Table S1. The number of foraging and tree trails was dependent on nest size, and this random effects regression model was used to compute residuals for individual nests in order to standardise trail number independently of nest size. The number of trails was log10-transformed (X+1).

| Random effects: |  |  |  |
| --- | --- | --- | --- |
| grouping variable | *b* | standard deviation | |
| nest ID (ICC = 0.001) | intercept | 0.01 |  |
|  | nest size | 0.13 |  |
|  | residual | 0.14 |  |
| Fixed effects: |  |  |  |
| variable | *b* (+/-95% CI) | *t* | *p* |
| intercept | -0.02 (-0.04, 0.003) | -1.67 | 0.10 |
| nest size | 0.21 (0.17, 0.24) | 10.71 | <0.001 |
| *N*_observations, nest ID_ = 505, 111 | |  |  |

Table S2. Exploratory analysis of potential predictors of the presence/absence of workers on tree trails using a generalized additive model. The objective of this model was to gauge the relative effect of temperature, light, wind, time of day and nest size on foraging activity.

| variable | EDF | *X*^2^ | *p* |
| --- | --- | --- | --- |
| surface temperature | 6.26 | 35.21 | < 0.001 |
| surface light | 6.42 | 14.10 | 0.06 |
| surface wind speed | 1.00 | 2.29 | 0.13 |
| time of day | 6.45 | 15.22 | 0.05 |
| nest size | 1.00 | 4.20 | 0.04 |
| nest ID (as factor) | 6.03 | 11.56 | 0.04 |
| adjusted *r*^2^ = 0.78, *N*_nests, observations_ = 11, 213 | | |  |

Table S3. Exploratory analysis of potential predictors of swarm activity using a generalized additive mixed model. The objective of this model was to gauge the relative effect of temperature, light, wind, time of day and nest size on swarm activity.

| variable | EDF | *F* | *p* |
| --- | --- | --- | --- |
| surface temperature | 4.74 | 9.76 | < 0.001 |
| surface light | 3.04 | 1.66 | 0.16 |
| surface wind speed | 1.00 | 0.05 | 0.82 |
| time of day | 3.34 | 9.44 | < 0.001 |
| nest size | 2.29 | 6.80 | < 0.001 |
| adjusted *r*^2^ = 0.54, *N*_nests, observations_ = 11, 127 | | |  |

Table S4. Summary statistics of light and wind alongside data on nest size for each nest used in the calculation of temperature-dependent performance curves (see also Figure S2).

|  |  | Summer |  | Winter |  |
| --- | --- | --- | --- | --- | --- |
|  |  | light  (μmol m^-2^ s^-1^) | wind  (km hr^-1^) | light  (μmol m^-2^ s^-1^) | wind  (km hr^-1^) |
| Nest | entrance holes | mean (range) | mean (range) | mean (range) | mean (range) |
| III | 39 | 949 (3 - 2169) |  | 502 (29 - 1355) | 1.4 (0 - 3.7) |
| LIX | 25 | 886 (14 - 2065) | 0.4 (0 - 1.9) | 467 (12 - 1238) | 0 (0 - 0) |
| LVI | 12 | 987 (34 - 2066) | 2.6 (0 - 16.6) | 325 (37 - 1056) | 0 (0 - 0) |
| LXI | 32 | 1104 (38 - 2114) | 3.9 (0 - 11.8) | 581 (39 - 1361) | 0.2 (0 - 1.6) |
| LXIV | 58 | 1182 (0.1 - 2333) | 2.9 (0 - 6.4) | 567 (25 - 1236) | 1.2 (0 - 6.1) |
| VII | 37 | 1108 (22 - 2051) | 1.6 (0 - 6.4) | 183 (24 - 1001) | 0 (0 - 0) |
| X | 6 | 517 (62 - 1619) | 3.6 (0 - 9.4) | 209 (71 - 412) | 1.3 (0 - 4.9) |
| XI | 42 | 1070 (59 - 2139) | 0.4 (0 - 3.2) | 630 (31 - 1306) | 0 (0 - 0) |
| XIV | 40 | 1232 (32 - 2303) |  | 596 (32 - 1155) | 2.2 (0 - 6.2) |
| XLIII | 31 | 1039 (3 - 2001) | 3.9 (0 - 17.2) | 105 (8 - 256) | 0 (0 - 0) |
| XVIII | 12 | 1194 (62 - 2087) | 3.1 (0 - 13.9) | 625 (56 - 1102) | 0 (0 - 0) |

Table S5. Emergence temperatures of ants induced to swarm based on a Bayesian change point analysis. Shown in (a) is the global or population level estimate, with the key parameter of interest being the computed change point temperature. This point corresponded to the minimum surface temperature of ant emergence for swarms, with the lower and upper bounds inferred by the quantiles of the highest-density intervals (HDI). R-hat is the Gelman-Rubin convergence diagnostic. Other parameter values correspond to the estimated swarm speed prior to emergence (which should converge on zero) and then at emergence, with the computed linear change in swarm speed increasing with temperature. Shown in (b) are the individual change point estimates for individual nests.

|  | variable | mean | lower HDI quantile | upper HDI quantile | R-hat | effective *N* |
| --- | --- | --- | --- | --- | --- | --- |
| a. population level | |  |  |  |  |  |
|  | change point (emergence surface temperature ^o^C) | 13.1 | 11.6 | 14.7 | 1.2 | 130 |
|  | change point SD | 4.44 | 1.93 | 7.20 | 1.1 | 599 |
|  | intercept, before (swarm speed mm s-1) | 0.99 | 0.10 | 1.90 | 1.0 | 350 |
|  | intercept, after (swarm speed mm s-1) | 9.93 | 8.67 | 11.20 | 1.1 | 451 |
|  | s | 2.97 | 2.67 | 3.30 | 1.0 | 3960 |
|  | slope, after (surface temperature ^o^C) | 0.14 | 0.09 | 0.20 | 1.0 | 771 |
| b. nest level | |  |  |  |  |  |
|  | LXI, change point (emergence surface temperature ^o^C) | 18.8 | 13.7 | 22.7 | 1.1 | 143 |
|  | LXIV, change point (emergence surface temperature ^o^C) | 11.1 | 7.0 | 14.5 | 1.0 | 932 |
|  | X, change point (emergence surface temperature ^o^C) | 15.0 | 11.6 | 18.8 | 1.0 | 1249 |
|  | XLIII, change point (emergence surface temperature ^o^C) | 14.3 | 11.5 | 18.3 | 1.1 | 943 |
|  | XVIII, change point (emergence surface temperature ^o^C) | 14.5 | 11.9 | 20.4 | 1.2 | 244 |
|  | LVI, change point (emergence surface temperature ^o^C) | 14.6 | 11.3 | 18.4 | 1.0 | 1214 |
|  | LIX, change point (emergence surface temperature ^o^C) | 14.4 | 12.6 | 16.1 | 1.2 | 145 |
|  | XCIII, change point (emergence surface temperature ^o^C) | 12.3 | 8.8 | 15.8 | 1.0 | 1361 |
|  | XIV, change point (emergence surface temperature ^o^C) | 9.0 | 5.1 | 12.7 | 1.0 | 951 |
|  | III, change point (emergence surface temperature ^o^C) | 6.8 | 4.7 | 8.7 | 1.1 | 227 |
|  | XI, change point (emergence surface temperature ^o^C) | 13.1 | 7.5 | 20.0 | 1.8 | 363 |

Table S6. Splines used to model longitudinal daily fluctuations in inferred hours available for foraging and nest defence (see Figure S4-S5). Differences among nests were evaluated by fitting alternative models with and without random effects for nest ID and applying restricted likelihood ratio tests (RLRT) to compare the fit of those models.

|  | model | RLRT | *p* |
| --- | --- | --- | --- |
| foraging activity (any) | |  |  |
|  | RLRT: random intercept + slope vs intercept only | 30.56 | < 0.001 |
|  | RLRT: random intercept vs no random effect | NA | NA |
| foraging activity (optimal) | |  |  |
|  | RLRT: random intercept + slope vs intercept only | 1.02 | 0.11 |
|  | RLRT: random intercept vs no random effect | 37.48 | < 0.001 |
| swarm activity (any) | |  |  |
|  | RLRT: random intercept + slope vs intercept only | 0.00 | 1.00 |
|  | RLRT: random intercept vs no random effect | 157.42 | < 0.001 |
| swarm activity (optimal) | |  |  |
|  | RLRT: random intercept + slope vs intercept only | 0.52 | 0.17 |
|  | RLRT: random intercept vs no random effect | 134.7 | < 0.001 |

Table S7. Formula used to convert measures taken with infra-red thermometer gun to values comparable to those taken with temperature probes positioned 1cm above nest surfaces. Probe type refers to those that recorded temperature only (coded 0) or temperature and humidity (1).

| variable | *β* | *t* | *p* |
| --- | --- | --- | --- |
| intercept | -7.69 | -12.71 | <0.001 |
| gun temperature^0.5^ | 6.19 | 52.89 | <0.001 |
| probe type | 0.60 | 0.69 | 0.49 |
| gun temperature^0.5^ * probe type | -0.38 | -2.14 | 0.03 |
| *N*_observations_ = 484 |  |  |  |

Figure S1. Details of the primary and adjusted performance curves estimated for foraging and swarm activity as a function of surface temperature. The adjusted performance took into account the statistical effects of nest size and time of day (see Table 1) and were benchmarked relative to the median nest size and 12 noon.

Figure S2. Companion data to Figure 1 showing (a) foraging and (b) swarm activity as a function of light, wind, time of day and nest size.

Figure S3. Details of (a) nest surface temperatures recorded over three weeks in mid-summer and mid-winter. These periods are highlighted by the dashed vertical lines in (b), which show the computed splines for the minimum and range in daily surface temperatures across the six focal nests. Gaps appear for nest XIV and XCIII because probes had been dislodged from the surface of the nest by animal (e.g., wombat) or bird (e.g., magpie) interference (e.g., see Figure S4).

Figure S4. Inferred daily hours available for (a) foraging or (b) nest defence based on performance curves shown in Figure 1 and the number of hours in which the surface temperature of the nest was within the lower and upper temperature thresholds tolerated by ants. Open circles are the number of inferred daily hours for activity. Trend lines are splines computed by the models reported in Table S6. Gaps in the data occurred because of battery failure or probes being displaced by kangaroos, wombats or birds.

Figure S5. Inferred daily hours available for (a) foraging or (b) nest defence reflecting the number of hours in which the surface temperature of the nest was within the optimal performance window corresponding to at least 90% of the maximum number of workers observed moving along tree trails or the maximum speed of swarms (dashed lines in Figure 1b, c). Open circles are the number of inferred daily hours for activity. Trend lines are splines computed by the models reported in Table S6. Gaps in the data occurred because of battery failure or probes being displaced by kangaroos, wombats or birds.

Figure S5. Continued.

Figure S6. Data used to compute the conversion equation reported in Table S1. This equation is shown here by the trend lines, colour coded by probe type.
